# Biotransformation of Scheelite CaWO_4_ by the Extreme Thermoacidophile *Metallosphaera sedula*: Tungsten–Microbial Interface

**DOI:** 10.3389/fmicb.2019.01492

**Published:** 2019-07-02

**Authors:** Amir Blazevic, Mihaela Albu, Stefan Mitsche, Simon K.-M. R. Rittmann, Gerlinde Habler, Tetyana Milojevic

**Affiliations:** ^1^Extremophiles/Space Biochemistry Group, Department of Biophysical Chemistry, University of Vienna, Vienna, Austria; ^2^Graz Centre for Electron Microscopy, Graz, Austria; ^3^Archaea Physiology and Biotechnology Group, Archaea Biology and Ecogenomics Division, Department of Ecogenomics and Systems Biology, University of Vienna, Vienna, Austria; ^4^Department of Lithospheric Research, University of Vienna, Vienna, Austria

**Keywords:** tungsten, *Metallosphaera sedula*, scheelite, analytical spectroscopy, nanoparticles

## Abstract

The tungsten–microbial interactions and microbial bioprocessing of tungsten ores, which are still underexplored, are the focus of the current study. Here we show that the biotransformation of tungsten mineral scheelite performed by the extreme thermoacidophile *Metallosphaera sedula* leads to the breakage of scheelite structure and subsequent tungsten solubilization. Total soluble tungsten is significantly higher in cultures containing *M. sedula* grown on scheelite than the abiotic control, indicating active bioleaching. Advanced analytical electron microscopy was used in order to achieve nanoscale resolution ultrastructural studies of *M. sedula* grown on tungsten bearing scheelite. In particular, we describe that *M. sedula* mediated the biotransformation of scheelite, which was accompanied by the release of tungsten into solution and tungsten biomineralization of the cell surface. Furthermore, we observed intracellular incorporation of redox heterogenous Mn- and Fe-containing nano-clusters. Our results highlight unique metallophilic life in hostile environments extending the knowledge of tungsten biogeochemistry. Based on these findings biohydrometallurgical processing of tungsten ores can be further explored. Importantly, biogenic tungsten carbide-like nanolayers described herein are potential targets for developing nanomaterial biotechnology.

## Introduction

A great variety of evolutionally diversified metallophilic microorganisms are equipped with unique capabilities and fascinating metabolic routes for manipulating minerals and dissolving them to access useful metals. The nature of the mineral–microbe interface, where electron and mass transfer processes arise, is a key element in the understanding of the transition of geochemistry to biochemistry. Consideration of the application of tungsten ores bioprocessing is restricted by the lack of research into tungsten–microbial interactions. Tungsten (W) is the only metal from the third transition series, which is shown to occur in biomolecules, where it is used in a few species of bacteria and methanogenic and hyperthermophilic archaea as an essential element, constitutive, and functional part of tungstopterin cofactor (Chan et al., 1995; Kletzin, 1996; Andreesen and Makdessi, 2008). Microbial assimilation of tungsten has been proposed in hydrothermal environments, where high concentrations of tungsten might be connected with metabolic activity of hyperthermophilic archaea (Holden and Adams, 2003). The recently shown passive tungstate biosorption by living or inactivated microbial cells has been suggested as an efficient strategy for tungsten recovery and recycling from aquatic systems (Malekzadeh et al., 2007; Takashi et al., 2013, 2016; Ogi et al., 2016). Hard, rare metal tungsten, with its extraordinary properties and the highest melting point of all metals is indispensible in modern high-tech industry. The currently used hydrometallurgical or pyrometallurgical processes enable the breakage of the tungsten–oxygen bond and release of the associated tungsten; however, the bioprocessing of tungsten bearing materials is not yet established.

The extreme thermoacidophile *Metallosphaera sedula* is a metal mobilizing archaeon, which thrives in hot acid (optimal growth at 73°C and pH 2.0) and utilizes energy from the oxidation of reduced inorganic sources, respiring aerobically on metals, molecular hydrogen, and reduced inorganic sulfur compounds (Huber et al., 1989; Auernik and Kelly, 2010; Wheaton et al., 2016; Kölbl et al., 2017). Up to now, the biooxidation capacity of *M. sedula* has merely been proposed as a procedure in the processing of sulfide and uranium ores (Auernik and Kelly, 2010; Mukherjee et al., 2012), but here we show that metal mobilizing archaeon *M. sedula* can grow on the non-sulfide tungsten ore scheelite. The elevated levels of tungsten mobilized from scheelite were detected in cultures of *M. sedula*, suggesting the mineral dissolution. To gain a close look into tungsten mineral–microbial interface during scheelite biotransformation performed by *M. sedula*, an integrative interdisciplinary approach, coupling analytical electron microscopy, analytical spectroscopy, wet chemistry, and microbiology, was applied. Here we report on the *M. sedula*-associated nanolayers of tungsten carbide-like material deposited over the microbial cell surface and we present its physicochemical characterization.

## Materials and Methods

### Strain, Media Composition and Cultivation Setup

*Metallosphaera sedula* (DSMZ 5348) cultures were grown aerobically in DSMZ88 *Sulfolobus* medium (initial pH of 2.0 with 10 N H_2_SO_4_ without further adjustments) as described previously (Kölbl et al., 2017). For chemolithoautotrophic growth cultures were supplemented with 10 g/liter scheelite mineral (average particle size 1 mm). Scheelite mineral concentrate containing impurities of Mn and Fe oxides (0.2 and 19.0% w/w, correspondingly) originated from the Serido Scheelite Province (Curais Novos, Rio Grande do Norte, Brazil) and was provided by Wolfram Bergbau und Hütten AG (St. Martin i.S., Austria). Scheelite was temperature sterilized at 180°C in a heating chamber for a minimum of 24 h prior to autoclavation at 121°C for 20 min. Abiotic controls consisting of uninoculated culture media supplemented with sterilized scheelite were included in all experiments. Growth of cells was monitored by phase contrast/epifluorescence microscopy and metal release. To visualize wiggling cells on solid particles, a modified DAPI (4′-6′-diamidino-2-phenylindole) staining was used (Huber et al., 1985); afterward the cells were observed and recorded with ProgRes^®^ MF cool camera (Jenoptik) mounted on Nikon eclipse 50i microscope, equipped with F36-500 Bandpass Filterset (ex, 377/50 nm; em, 447/60 nm).

### Metal Analysis

To determine the extracellular concentrations of metal ions mobilized from scheelite, culture samples were clarified by centrifugation. Samples of the resulting supernatants were filtered (0.44 μm pore size) and analyzed by inductively coupled plasma-optical emission spectrometer (ICP-OES) Perkin Elmer Optima 5300 DV. All reported values are averages from triplicate samples.

### Wavelength Dispersive X-Ray Fluorescence Analysis (WD-XRF)

Scheelite was analyzed using Wavelength dispersive X-ray fluorescence (WD-XRF) spectrometer Zetium PW 5400, Panalytical and SuperQ-program. The evaluation of the measuring data was done using Uniquant program and thereby the chemical composition was ascertained. In parallel the loss of ignition of the sample was determined at 1000°C.

### Scanning Electron Microscopy With Energy Dispersive Spectroscopy (SEM/EDS)

Cultures of *M. sedula* autotrophically grown on scheelite were harvested after 21 days of cultivation, spread evenly on glass plates (Ø 7 cm, VWR International), and dehydrated within 30 days under oxic conditions at room temperature. Abiotic controls consisting of uninoculated culture media were included in all the experiments. The morphology of the dehydrated cells of *M. sedula* was examined with a Zeiss Supra 55 VP scanning electron microscopy (SEM) as described previously (Kölbl et al., 2017).

### Focused Ion Beam (FIB) Sample Preparation

Sample preparation for transmission electron microscopy (TEM) has been performed by focused ion beam (FIB) sputtering using a FEI Quanta 3D FEG instrument, equipped with an electron column hosting a field-emission electron source (Schottky emitter) and an ion column hosting a Ga-liquid metal ion source (LMIS). Sputtering progress has been monitored by electron beam (EB) induced secondary electron (SE) imaging at EB settings of 5 keV accelerating voltage, using standard mode spot number 3.5 and a 30 μm SEM aperture as probe current settings (yielding approximately 15 pA probe current). Before sputtering, a Pt layer (length × width × height = 8 × 3 × 3 μm) was deposited onto the cells of *M. sedula* by applying FIB Pt deposition at 16 kV IB acceleration voltage and 50 pA IB current for 5 min at the start, and continued after increasing IB current to 150 pA. The deposited nanocrystalline Pt served as protection layer during subsequent preparation steps. Using 30 kV FIB accelerating voltage and 7 nA IB current regular cross sections have been sputtered at both sides of the Pt top layer while the beam incidence was perpendicular to the substrate surface. All sections were performed with IB scanning directed toward the TEM foil, which represented the end point of each sputtering step. Before foil extraction from the sample, two cleaning cross sections have been sputtered at each side of the foil using 30 kV IB accelerating voltage, and IB currents of 5 and 1 nA, respectively. The 3-μm thick foil has been transferred to an Omniprobe Cu TEM grid using an Omniprobe 100.7 micromanipulator for *in situ* lift-out. The sample has been temporarily mounted to a W-needle, and finally attached to the Cu TEM grid by Pt deposition at IB 30 kV and an IB probe current 100 pA. After the foil transfer to the TEM grid, several final thinning steps by cleaning cross sections have been performed alternately at both foil surfaces using successively lower IB probe current (300, 100, 50, and 10 pA) at successively smaller IB incidence angles (±1.5°, 1.2°, and 1°, respectively). The beam incidence direction therefore was close to parallel to the foil plane. Between each sputtering step the stage has been rotated by 180° in order to monitor the foil surface during sputtering and in order to achieve a perfectly central cut through the cells by reaching the maximum cell diameters. SE image contrast between the Pt top layer (higher signal intensity) and the cells (lower signal intensity) allowed monitoring the sputtering sites and the cells behavior during sputtering. Only a window area of the foil was completely finally thinned, whereas marginal parts of the foil had been left thicker to form a stabilizing frame. As the scheelite-substrate of the cells behaved significantly weaker than the Pt top layer and the cells, a hole was generated in the substrate below the cells during the last final thinning steps. The obtained thinned foil area has finally been thinned to 55–65 nm thickness measured at the Pt top layer, but was slightly thinner in areas below the Pt top layer. The FIB preparation procedure led to the reduction of the maximum cell diameters, while Pt-deposition caused some flattening of the initially spheroidal cells. On the other hand, a slight off-center position of the cell cross-section could also lead to a reduction in the cross-section area of the cells.

### TEM Imaging and EDS Investigations

High resolution STEM investigations were performed on a probe corrected FEI Titan G2 60–300 (S/TEM) microscope with an X-FEG Schottky field-emission electron source operated at 300 kV (current of 150 pA, beam diameter of 1 Å). The microscope is equipped with a FEI Super-X detector (Chemi-STEM technology), consisting of four separate silicon drift detectors and a Dual EELS – Gatan Imaging Filter (GIF) Quantum. High Angular Annular Dark Field (HAADF) and Annular Dark Field (ADF) detectors were used to acquire micrographs. Conventional and analytical TEM was performed using a FEI Tecnai F20 microscope operated at 200 kV under cryo conditions. The microscope is suited with a Schottky emitter, a monochromator, a post-column high resolution GIF and a Si(Li) X-ray detector with ultrathin window. Energy filtered transmission electron microscopy (EFTEM), scanning transmission electron microscopy (STEM) and analytical spectroscopy by using electron energy loss (EELS) and dispersive X-ray (EDS) were carried out for different areas of *M. sedula* cells. For each area jump ratio images of the following elements: W, P, S, C, N, Mn, and Fe as well as EELS and EDS spectra from representative nano-particles on the cell surface and inside the cells have been acquired. EDS spectra were recorded in STEM mode, with a specimen diameter of about 1 nm. The images and spectra were processed with Gatan’s Digital Micrograph being corrected for dark current and gain variations. Element quantification for both EELS and EDS spectra was performed by using the k-factor method (Hofer, 1991; Hofer and Kothleitner, 1993; Albu et al., 2016).

## Results and Discussion

### Biotransformation of the Tungsten Mineral Scheelite (CaWO_4_)

**FIGURE 1:**
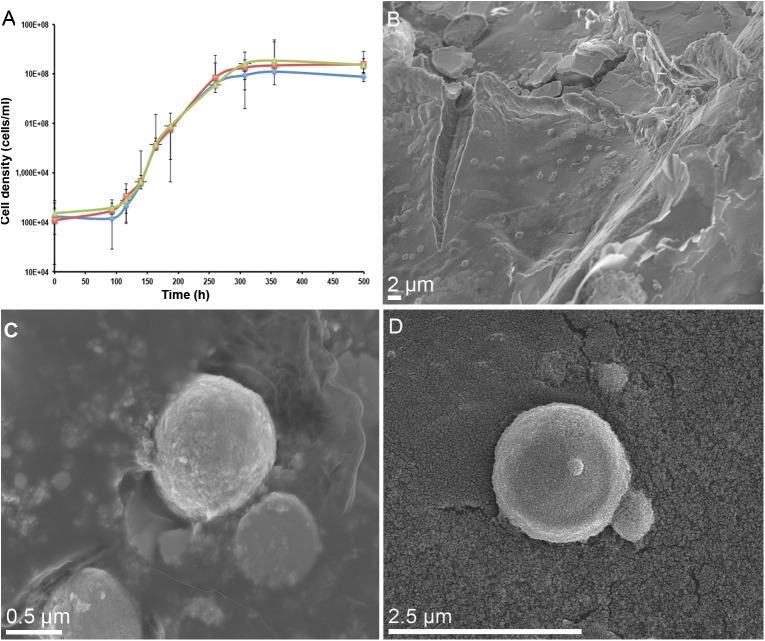
Biotransformation of the tungsten mineral scheelite (CaWO_4_) by *M. sedula*. **(A)** Growth curves of autotrophic cultures of *M. sedula* cultivated at 73°C on scheelite. Biological replicates are indicated in green, red, and blue colors. **(B)** Scanning electron microscopy (SEM) image showing cells of *M. sedula* colonizing the surface of scheelite. **(C,D)** Higher magnification SEM images showing attached single cells of *M. sedula*. Points and error bars show the mean and error-represented standard deviation, respectively, of *n* = 3 biological replicates. If not visible, error bars are smaller than symbols.

**FIGURE 2:**
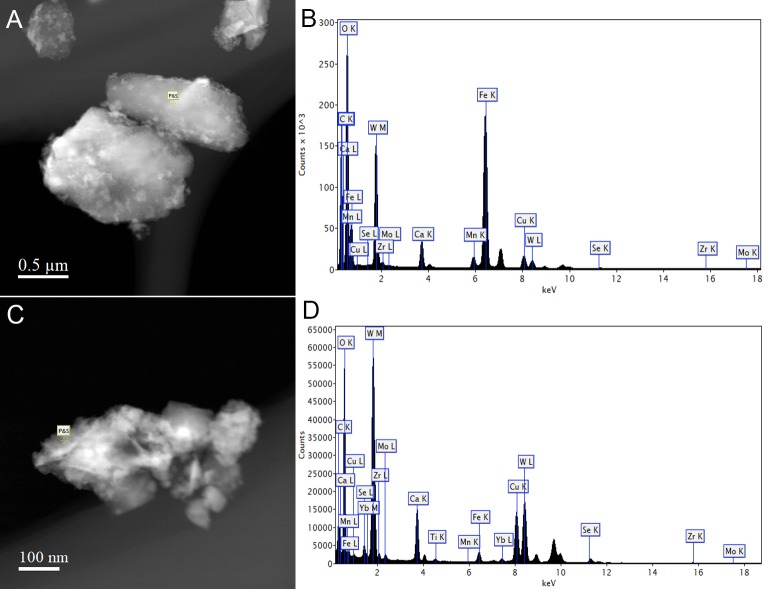
Scanning transmission electron microscopy-energy-dispersive X-ray spectroscopy (STEM-EDS) point analysis of the chemical composition of scheelite used in the study. Representative STEM images **(A,C)** and corresponding EDS spectra **(B,D)** are shown, indicating that CaWO_4_ contained impurities of iron and traces of other elements.

**FIGURE 3:**
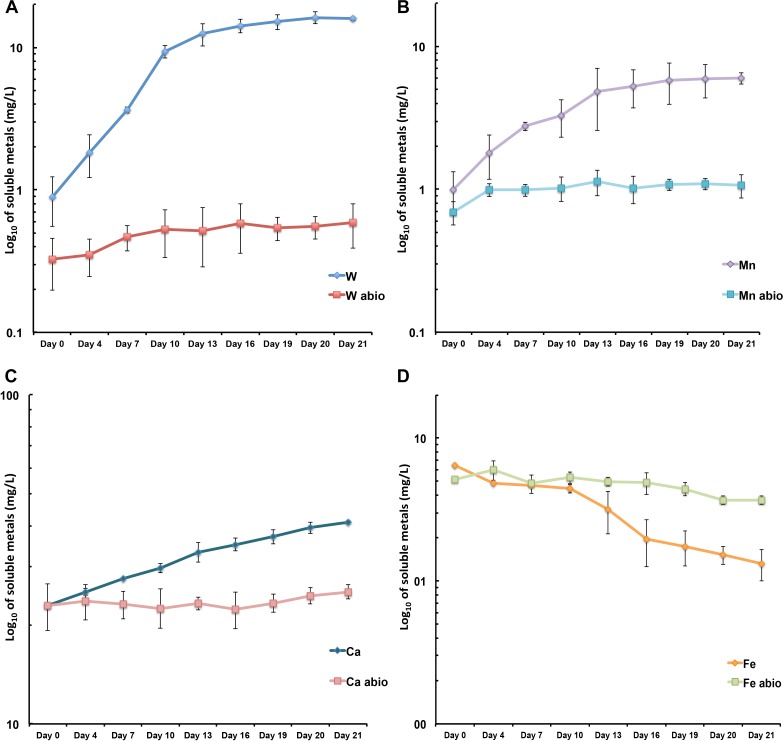
Scheelite bioleaching by *M. sedula*. **(A–D)** ICP-OES analysis of released metal ions in supernatant of *M. sedula* cultures. Samples were taken from cultures of *M. sedula* and corresponding abiotic controls (abio). Total soluble metals are represented as log_10_ values (mg/liter), measured as a function of hours postinoculation. Points and error bars show the mean and error-represented standard deviation, respectively, of *n* = 3 biological replicates. If not visible, error bars are smaller than symbols. Legends represent metal ion measured.

*Metallosphaera sedula* was aerobically cultivated on a calcium tungstate mineral scheelite at 73°C with CO_2_ supplementation (Figure 1A, Supplementary Figure S1, and Supplementary Video S1). Scheelite has a vitreous to adamantine luster, the mineral’s Mohs hardness of 4.5–5; specific gravity of 5.9–6.1; and tetragonal crystal system (Anthony et al., 1990). The chemical composition of scheelite used in the study was examined by point analysis of nanometer scale particles using STEM-EDS technique. Representative images and corresponding EDS spectra are shown on Figure 2, indicating that CaWO_4_ contained impurities of iron and traces of other elements, e.g., Mn, Zr, Se, Ti, and Mo (Figure 2B,D). Furthermore, evaluation of bulk elemental composition of scheelite used in the study was performed by WD-XRF analysis (Table 1). WD-XRF technique showed W, Fe, Ca, Ti, and Si as major elements and Al, Mn, Zr, Mo, Na, Mg, P, S, Cl, K, V, Cr, Mn, Co, Cu, Zn Ga, Se, Sr, Y, Sn, Ba, Pb, and Bi as trace elements (Table 1). *M. sedula* was capable of lithoautotrophic growth on scheelite as the sole energy source (Figure 1A, Supplementary Figure S1, and Supplementary Video S1). Figure 1A shows the growth curves of *M. sedula* exposed to scheelite, reaching cell densities in excess of 10 × 10^8^ cells/mL. A wiggling of the scheelite-colonizing cells of *M. sedula* was observed and recorded after visualization by epifluorescence microscopy (Supplementary Video S1). Together with pilus-like structures, these typical wiggling activities along the metal ore have been previously described for *M. sedula* grown on iron-and sulfur-bearing mineral substrates (Huber et al., 1989). We further examined the interaction of cells with the mineral particles and bioleaching capacities of *M. sedula* grown on scheelite. After 21 days of CO_2_-supplemented cultivation on scheelite, the growth medium (leachate solution) was analyzed for metal content. Analysis of metal elements mobilized from scheelite was performed using Inductively coupled plasmon resonance spectroscopy coupled to optical emission spectroscopy (ICP-OES). At 21 days postinoculation, ICP-OES analysis showed elevated levels of released W (Figure 3A) and Mn (Figure 3B) along with a moderate increase of Ca (Figure 3C) in cultures of *M. sedula*. Total soluble W and Mn were significantly higher in cultures containing *M. sedula* grown on scheelite than the corresponding abiotic controls, indicating bioleaching process (Figure 3A,B). Additionally, a decrease of the Fe level was observed in leachate solution when compared to the corresponding abiotic control (Figure 3D), which can be possibly attributed to the formation of insoluble Fe-rich precipitates (e.g., iron oxyhydroxides) and/or microbial consumption of Fe. Studies with SEM showed that *M. sedula* colonizes scheelite, indicating cell attachment to the mineral surface. Images of attached cells of *M. sedula* on scheelite surfaces are shown in Figure 1B–D (for abiotic scheelite surface see Supplementary Figure S2), suggesting the “contact” mechanism of microbial-mediated mineral biotransformation, which implies that mineral dissolution occurs at the interface between cell wall and the mineral surface (Rohwerder et al., 2003). The cells with budding vesicles occurred in this case as well, along with precipitated nanoglobules attached to the surface of *M. sedula* cells (Figure 1D). Along with scheelite-attached cells, the planktonic cells of *M. sedula* were, as well, clearly observed.

**Table 1:** Wavelength dispersive X-ray fluorescence analysis (WD-XRF) of scheelite used in the study.

Element name	Wt%
Na	0.03
Mg	0.2
Al	0.9
Si	3.5
P	0.03
S	0.8
Cl	0.04
K	0.02
Ca	13.7
Ti	1.2
V	0.05
Cr	0.05
Mn	0.2
Fe	22.6
Co	0.01
Cu	0.06
Zn	0.02
Ga	0.02
Se	0.09
Sr	0.02
Y	0.01
Zr	0.8
Mo	0.8
Sn	0.02
Ba	0.1
W	54.2
Pb	0.04
Bi	0.1
L.O.I. at 1000°C	0.3
SUM	99.9

### Elemental Ultrastructural Analysis of Scheelite-Grown Cells of *M. sedula*

**FIGURE 4:**
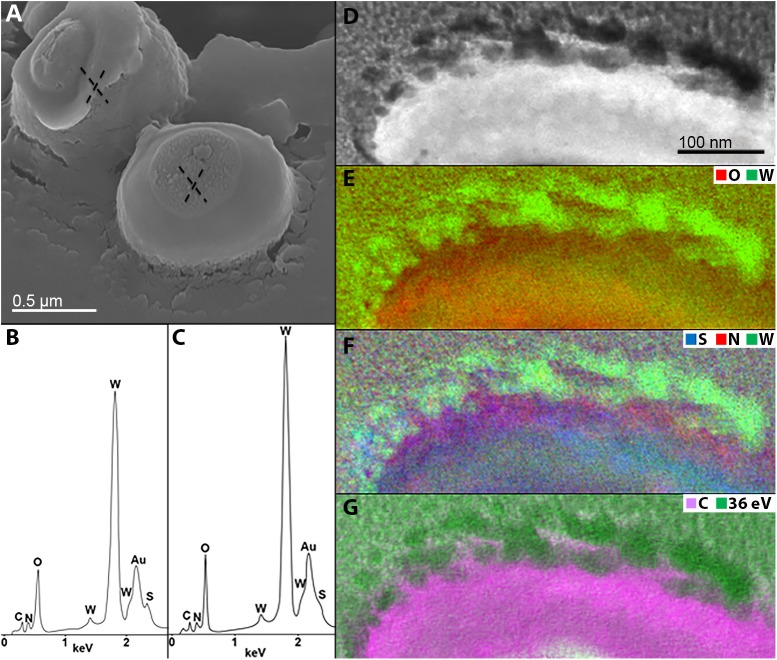
Elemental ultrastructural analysis of scheelite-grown cells of *M. sedula*. **(A)** SEM image showing cells of *M. sedula* deposited over scheelite surface. **(B,C)** Corresponding EDS spectra taken in regions marked in panel **(A)**. Au peaks in EDS spectra are due to sample coating with an Au layer; full length EDS spectra are represented in Supplementary Figure S3. **(D)** Scanning transmission electron microscopy (STEM) image of a cell fragment of *M. sedula* used for energy-filtered transmission electron microscopy (EFTEM) analysis. **(E–G)** Corresponding tungsten (W), oxygen (O), sulfur (S), nitrogen (N) and carbon (C) elemental maps. W-post-edge energy filtered at 36 eV is represented delivering the best image contrast.

To improve our understanding of microbial-mediated scheelite dissolution, the surface of scheelite-grown *M. sedula* was examined with SEM coupled with Energy Dispersive Spectroscopy (SEM/EDS) analysis. Inorganic ions relieved in course of scheelite biotransformation tend to accumulate on the surface of *M. sedula* cells, forming mineral phase precipitates on its surface-layer (S-layer). Various mineral precipitates were reported earlier on the S-layer of other biomineralizing extremophilic archaea and bacteria (Orange et al., 2011, 2014; Oggerin et al., 2013; Sánchez-Román et al., 2015; Kish et al., 2016). The EDS spectrum of mineralized cells of *M. sedula* displayed strong dominated W peak along with less intensive peaks of O, C, and N. Distinguishable signals for S were detected in the cells as well (Figure 4A–C and Supplementary Figure S3). To further characterize microbial–mineral interface, we performed ultrastructural analysis of cells of *M. sedula* grown on scheelite. The mineralized cells of *M. sedula* grown on scheelite represented a kind of hard and brittle biological material that is difficult to cut with a diamond knife in conventional ultramicrotome procedures. Therefore, in order to overcome this limiting factor, a Focused Ion Beam (FIB-SEM) was applied to prepare foil samples for the analysis at high resolution (see Supplementary Figure S4). Further elemental ultrastructural analysis enabled us to verify the content and localization of metals in *M. sedula* (Figure 4D–G). The following observations were made by using energy-dispersive X-ray spectroscopy (EDS) analysis performed in STEM mode: (1) the elemental maps showed abundant W content on the cell surface of *M. sedula*; (2) W was selectively deposited on the cell surface massively encrusting *M. sedula* cells; (3) carbon and oxygen were evenly represented giving strong intracellular signals which likely arose from organic content (e.g., proteins and carbohydrates) present in *M. sedula* cells; (4) N signal was more pronounced in the area of membrane localization; (5) weak signals of S were detected in the close proximity to the inner part of cell envelope. These observations are in agreement with our microscopy SEM-EDS results, which also indicated W, O, C, N, and S content in *M. sedula* cells (Figure 4B,C).

### Tungsten Deposits on the Cell Surface of *M. sedula*

**FIGURE 5:**
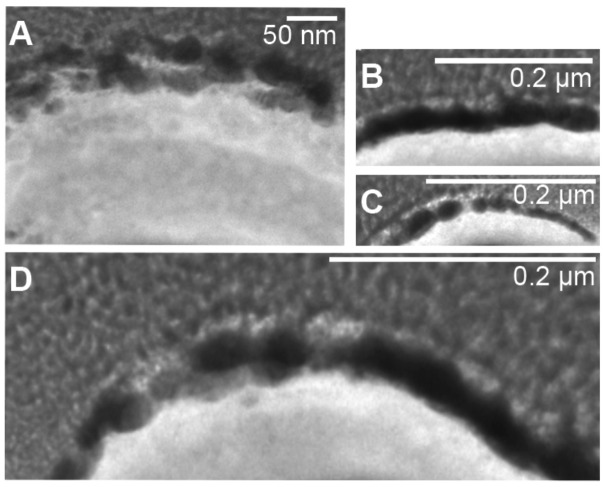
Fragments of S-layer of cells of *M. sedula* heavily encrusted with tungsten. **(A–D)** W post-edge energy filtered transmission electron microscopy (EFTEM) images of different cell fragments of cells of *M. sedula* grown on scheelite.

**FIGURE 6:**
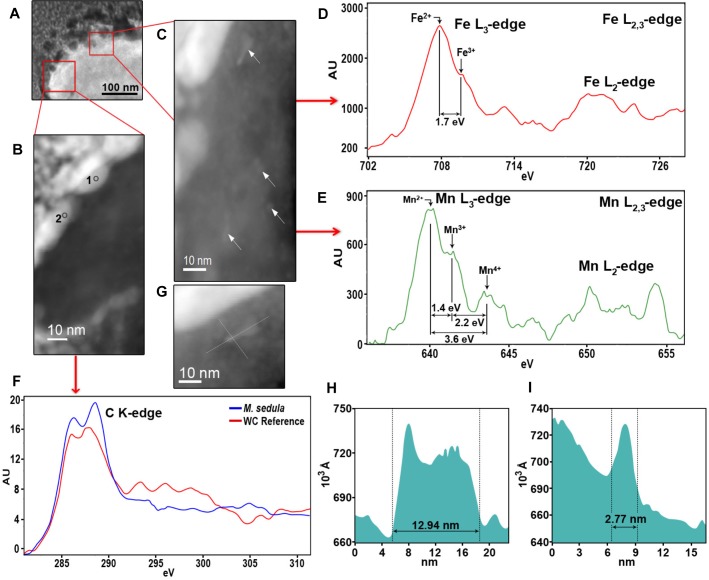
Nanoanalytical spectroscopy investigations of *M. sedula* grown on scheelite. **(A)** STEM image of the whole cell fragment of *M. sedula* used for electron energy loss spectra (EELS) analysis. **(B)** The inset with magnified HAADF-STEM image of tungsten encrusted cell surface of *M. sedula* depicted in panel **(A)** and used for EELS measurements. **(C)** The inset with magnified HAADF-STEM image of intracellular part with nanoparticles depicted in panel **(A)** and used for EELS measurements. **(D,E)** Corresponding representative Fe and Mn L_2,3_-edge core electron energy loss (EEL) spectra acquired from areas depicted with arrows in panel **(C)**. **(F)** Representative C K-edge EEL spectra acquired from the W-encrusted cell surface layer of *M. sedula* in areas depicted in panel **(B)** and tungsten carbide reference. **(F)** A magnified HAADF-STEM image showing intracellular Mn-, Fe-nanoclusters. **(H,I)** The particles size distribution plots, representing the size of Mn-, Fe-bearing intracellular nanoclusters shown in panels **(C,G)**.

A tungsten-encrusted layer was formed around the cells of *M. sedula* (Figure 4D–G, 5). This biomineralized layer was approximately 100 nm thick and it appeared to comprise a randomly deposited assembly of W-bearing globules of variable size, which massively encrust the surface area of the cells of *M. sedula* (Figure 5). Due to its pronounced thickness and good contrast, the tungsten-containing layer was visible in both the HAADF scanning TEM (STEM) and energy filtered TEM (EFTEM) images (Figure 4–6). The EELS spectra in Figure 6 acquired from the tungsten-encrusted cell surface layer, showed the presence of a carbon K-edge at 284 eV, which is similar to the carbon K-edge of a tungsten carbide (WC) reference in terms of both edge energy position and fine structure features (Figure 6F). Hence, according to our observations, the total amount of mobilized W from scheelite did not only comprise the soluble released W measured by ICP-OES in culture medium of *M. sedula* (Figure 3A), but also W in biogenic tungsten encrustation of the cell surface (Figure 4D–G, 5, 6A–C).

**FIGURE 7:**
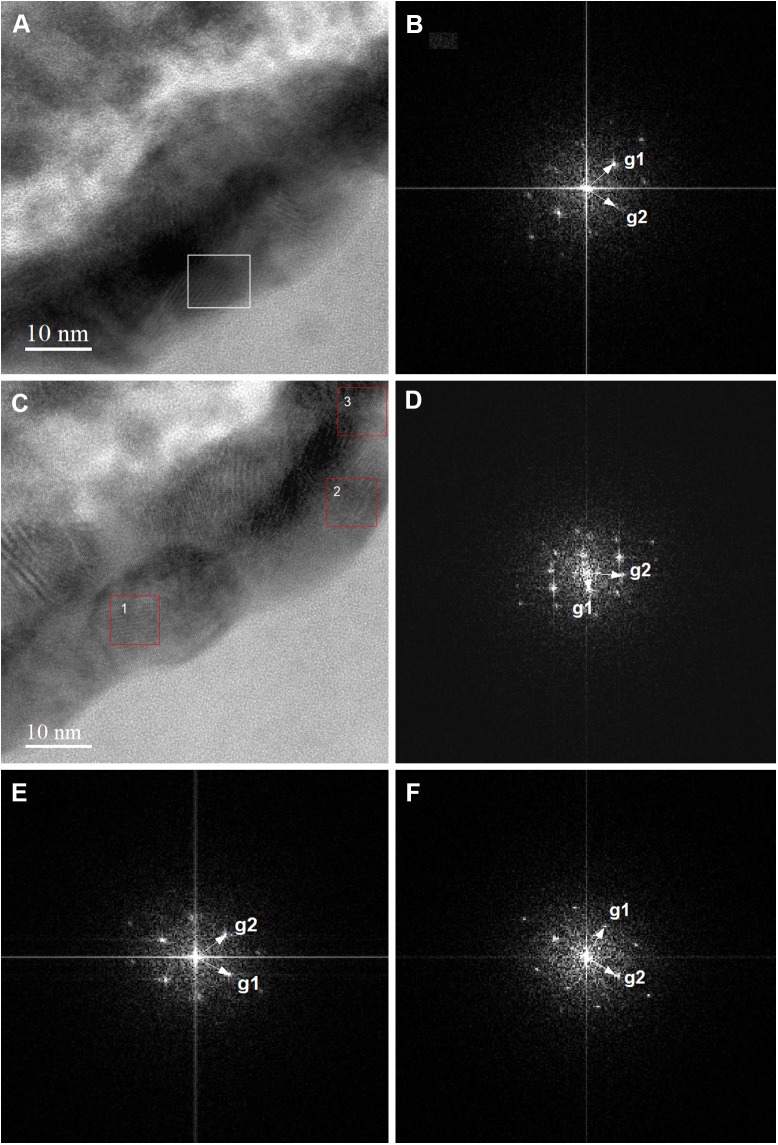
High-resolution TEM (HR-TEM) and Fast Fourier Transforms (FFTs) of biogenic tungsten deposits on the cell surface of *M. sedula* grown on scheelite. **(A,C)** High-resolution TEM (HR-TEM) images of S-layer fragments of *M. sedula* cell. **(B)** Fast Fourier Transform (FFT) acquired from the tungsten crystalline cell surface deposits square-labeled in panel **(A)**. **(D–F)** FFTs obtained from the tungsten crystalline cell surface deposits square-labeled in panel (1–3, correspondingly) **(C)**.

Furthermore, the S-layer of *M. sedula* cells was further examined by high-resolution TEM (HR-TEM) performed on thin foils (ca. 60 nm thickness) preliminary produced by FIB milling (Supplementary Figure S4). HR-TEM analysis revealed a crystalline microstructure of tungsten-bearing deposits over the S-layer with lattice parameters closed to different tungsten carbide structures [hexagonal WC: *a* = 0.28946 nm, *b* = 0.28946 nm, *c* = 0.28576416 nm (Page et al., 2008) and trigonal W_2_C *a* = 0.5188 nm, *b* = 0.5188 nm, *c* = 0.47273 nm (Kurlov and Gusev, 2007)] (Figure 7). Similar tungsten carbide-like structures we have reported previously for *M. sedula* cells cultivated with another tungsten-bearing material (Milojevic et al., 2019).

### Detection of Intracellular Mn, Fe-Nanoclusters in Cells of *M. sedula* Grown on Scheelite

HAADF-STEM imaging of fine intracellular structure, which produces image contrast based on atomic number (Z-contrast), revealed the presence of nanosized particles of Mn and Fe content (Figure 6). The EELS measurements acquired locally (point analysis with a beam diameter of 1 nm) in STEM mode demonstrated that these metal nano-clusters are bearing a mixed valence iron and manganese distribution with the dominant Fe^2+^ and Mn^2+^ species (Figure 6D,E). The diameters of the redox heterogenous nanoparticles ranged from 3 to 13 nm, as shown in Figure 6G,F,I, and they were evenly distributed in the cytoplasm of *M. sedula* (Figure 6C). Iron and manganese represented in mixed valence states in these nanoparticles might offer the metal ions of a suitable redox potential (Fe^2+^ and Mn^2+^) which are accessible to *M. sedula* as energy source. The observed Mn, Fe-bearing intracellular nano-inclusions most likely originate from Mn and Fe trace impurities in scheelite and represent clusters of metal ions with redox potential appropriate for biooxidative metabolic activity of *M. sedula*. These Fe, Mn-bearing nano-inclusions may provide reduced metallic species (Fe^2+^ and Mn^2+^) deposited inside the cell as a storage depot, which can be used by *M. sedula* to satisfy energy demand for respiration needs. However, at this stage of this analysis, the identification of the actual energy source (or sources) for growth and a correlation of a sufficient quantity of that oxidizable source to support the observed quantity of growth cannot be confirmed. It is possible to propose that the *M. sedula* mediated biooxidation of iron and manganese destroyed the scheelite structure, leading to the release of W into solution. The cells of *M. sedula* are enveloped by a highly ordered crystalline proteinaceous S-layer (Veith et al., 2009), enriched with negatively charged carboxyl groups, which are prone to sorption of metal ions (Banfield et al., 2000; Phoenix et al., 2002; Lalonde et al., 2008; Orange et al., 2011; Westall and Cavalazzi, 2011) and are frequently preferential locations for the nucleation of minerals that might subsequently grow upon crystallization (Banfield et al., 2000). A certain portion of W ions released in the leachate solution may tend to interact with the COOH groups on S-layer of the cells of *M. sedula*, forming structures that closely resemble tungsten carbide-like structures. This in turn leads to the subsequent biomineralization of cell surface of *M. sedula* and eventually results to its encapsulation with tungsten. The possibility of specific molecular interactions between released W ions and the membrane-associated molecular machinery of *M. sedula* cannot be ruled out. The genome of *M. sedula* encodes a specific set of putative tungsten binding and transporting proteins (Msed_1780, Msed_1781, and Msed_1613) (Wheaton et al., 2016). The annotated W transporter Msed_1780 was shown to be upregulated 10- to 20-fold in response to metals shock in a comprehensive transcriptomic analysis published earlier (Wheaton et al., 2016). Additionally, W ions released from scheelite potentially can also be consumed for a biosynthesis of W cofactor-containing enzymes in *M. sedula* (e.g., formate dehydrogenase subunit alpha Msed_1457) (Wheaton et al., 2016). In this regard, the molecular machinery of *M. sedula* responsible for tungsten binding, acquisition and assimilation is a topic that deserves more attention and thorough analysis in the future. Our results do not unravel the exact mechanism of the formation of tungsten carbide-like membrane-associated structures, but do indicate that *M. sedula* forms tungsten-bearing mineralized S-layer via encrusting with tungsten carbide-like compounds. Further advanced synchrotron-assisted spectroscopic studies of the nanoscale interface between scheelite and *M. sedula* could certainly clarify the redox route of released tungsten. μ-XANES techniques can be used in order to determine the nature of W environment in these biogenic deposits.

## Conclusion

In conclusion, we have shown the biotransformation of tungsten mineral scheelite by *M. sedula* accompanied by (1) tungsten solubilization in the leachate solution and (2) biomineralization of *M. sedula* S-layer with crystalline nanoparticles containing carbide-like tungsten. This phenomenon can be attributed to the highly ordered proteinaceous S-layer of *M. sedula* which promoted the sorption of the metal ions serving as nucleation centers for crystallization and therefore facilitating the further growth of the nanoparticles. Moreover, the intracellular inclusions of Mn and Fe of redox heterogenous nature can represent a nanometer sized energy storage depot for *M. sedula* cells. It is possible to propose that the *M. sedula* mediated biooxidation of iron and manganese destroyed the scheelite structure, leading to the release of W into solution. This part of proposed scenario may constitute a direct mechanism of mineral breakage. In addition to the direct *M. sedula* mediated biooxidation of metals, the involvement of abiotic factors may very well facilitate the process of tungsten mobilization from scheelite, representing an indirect mechanism of metal solubilization. Abiotic oxidizing attack of Fe^3+^ on the solid mineral enables the mobilization of metals from the solid matrix. Similarly, the results recently obtained by Wheaton et al. (2019) suggest that both direct and indirect mechanisms contribute to the dissolution of metal oxides and the mobilization of molybdenum and vanadium mediated by *M. sedula*. Apart from this, dissolution of scheelite under acidic conditions promotes calcium precipitation, which in turn can dissolve solid mineral phase. Most likely, the combination of these two biogenic and abiotic factors results to the observed mobilization of tungsten from the solid mineral matrix of scheelite.

This study provides information concerning the possible role of microorganisms in natural environments enriched with tungsten and corresponding microbial fingerprints, thus helping to unravel the biogeochemistry of tungsten. The findings presented herein will be useful for currently underrepresented and less studied biomining of tungsten ores, where biooxidative dissolution pre-treatment might be useful. Further, biogenic tungsten carbide-like nanostructures described herein represent a potential sustainable nanomaterial obtained by the environmentally friendly microbial-assisted design. These nanoparticles of carbide-like tungsten can find a wide application range from alloys and nanocomposites to corrosion-resistant coatings and hard metals manufacturing (Kear and McCandlish, 1993; Goren-Muginstein et al., 1998; Garcia-Esparza et al., 2013; Kang et al., 2017).

## Data Availability

All datasets generated for this study are included in the manuscript and/or the Supplementary Files.

## Author Contributions

All authors performed experiments, provided editorial input, made substantial contributions to the acquisition, analysis, and interpretation of data described in this article, critically reviewed the report, and approved the final version.

## Conflict of Interest Statement

The authors declare that the research was conducted in the absence of any commercial or financial relationships that could be construed as a potential conflict of interest.

## References

[B1] Albu M. Pal A. Gspan C. Picu R. C. Hofer F. Kothleitner G. (2016). Self-organized Sr leads to solid state twinning in nano-scaled eutectic Si phase. *Sci. Rep.* 6:31635. 10.1038/srep31635 27527789 PMC4985832

[B2] Andreesen J. R. Makdessi K. (2008). Tungsten, the surprisingly positively acting heavy metal element for prokaryotes. *Ann. N. Y. Acad. Sci.* 1125 215–229. 10.1196/annals.1419.003 18096847

[B3] Anthony J. W. Bideaux R. A. Bladh K. W. Nichols M. C. (1990). *Handbook of Mineralogy.* Tucson, AZ: Mineral Data Publishing.

[B4] Auernik K. S. Kelly R. M. (2010). Physiological versatility of the extremely thermoacidophilic archaeon *Metallosphaera sedula* supported by transcriptomic analysis of heterotrophic, autotrophic, and mixotrophic growth. *Appl. Environ. Microbiol.* 76 931–935. 10.1128/AEM.01336-09 20008169 PMC2813022

[B5] Banfield J. F. Welch S. A. Zhang H. Ebert T. T. Penn R. L. (2000). Aggregation-based crystal growth and microstructure development in natural iron oxyhydroxide biomineralization products. *Science* 289 751–754. 10.1126/science.289.5480.751 10926531

[B6] Chan M. K. Mukund S. Kletzin A. Adams M. W. Rees D. C. (1995). Structure of a hyperthermophilic tungstopterin enzyme, aldehyde ferredoxin oxidoreductase. *Science* 267 1463–1469. 10.1126/science.7878465 7878465

[B7] Garcia-Esparza A. T. Cha D. Ou Y. Kubota J. Domen K. Takanabe K. (2013). Tungsten carbide nanoparticles as efficient cocatalysts for photocatalytic overall water splitting. *ChemSusChem* 6 168–181. 10.1002/cssc.201200780 23255471

[B8] Goren-Muginstein G. R. Berger S. Rosen A. (1998). Sintering study of nanocrystalline tungsten carbide powders. *Nanostruct. Mater.* 10 795–804. 10.1016/s0965-9773(98)00116-0

[B9] Hofer F. (1991). Determination of inner-shell cross-sections for EELS-quantification. *Microsc. Microanal. Microstruct.* 2 215–230. 10.1051/mmm:0199100202-3021500

[B10] Hofer F. Kothleitner G. (1993). Quantitative microanalysis using electron energy-loss spectrometry. I. Li and Be in oxides. *Microsc. Microanal. Microstruct.* 4 539–560. 10.1051/mmm:0199300406053900

[B11] Holden J. F. Adams M. W. (2003). Microbe–metal interactions in marine hydrothermal environments. *Curr. Opin. Chem. Biol.* 7 160–165. 10.1016/s1367-5931(03)00026-7 12714047

[B12] Huber G. Spinnler C. Gambacorta A. Stetter K. O. (1989). Metallosphaera sedula gen. and sp. nov. represents a new genus of aerobic, metal-mobilizing, *Thermoacidophilic archaebacteria*. *Syst. Appl. Microbiol.* 12 38–47. 10.1016/S0723-2020(85)80021-7

[B13] Huber H. Huber G. Stetter K. O. (1985). A modified DAPI fluorescence staining procedure suitable for the visualization of lithotrophic bacteria. *Syst. Appl. Microbiol.* 6 105–106. 10.1016/s0723-2020(85)80021-7

[B14] Kang J. S. Kim J. Lee M. J. Son Y. J. Jeong J. Chung D. Y. (2017). Electrochemical synthesis of nanoporous tungsten carbide and its application as electrocatalysts for photoelectrochemical cells. *Nanoscale* 17 5413–5424. 10.1039/C7NR00216E 28300257

[B15] Kear B. H. McCandlish L. E. (1993). Chemical processing and properties of nanostructured WC-Co materials. *Nanostruct. Mater* 3 19–30. 10.1016/0965-9773(93)90059-k

[B16] Kish A. Miot J. Lombard C. Guigner J. M. Bernard S. Zirah S. (2016). Preservation of archaeal surface layer structure during mineralization. *Sci. Rep.* 6:26152. 10.1038/srep26152 27221593 PMC4879539

[B17] Kletzin A. (1996). Tungsten in biological systems. *FEMS Microbiol. Rev.* 18 5–63. 10.1016/0168-6445(95)00025-9 8672295

[B18] Kölbl D. Pignitter M. Somoza V. Schimak M. P. Strbak O. Blazevic A. (2017). Exploring fingerprints of the extreme thermoacidophile metallosphaera sedula grown on synthetic martian regolith materials as the sole energy sources. *Front. Microbiol.* 8:1918. 10.3389/fmicb.2017.01918 29062303 PMC5640722

[B19] Kurlov A. S. Gusev A. I. (2007). Neutron and x-ray diffraction study and symmetry analysis of phase transformations in lower tungsten carbide W2C. *Phys. Rev. B.* 76:174115 10.1103/PhysRevB.76.174115

[B20] Lalonde S. V. Smith D. S. Owttrim G. W. Konhauser K. O. (2008). Acid-base properties of cyanobacterial surfaces. *Geochim. Cosmochim. Acta* 72 1257–1268. 10.1016/j.gca.2007.10.031

[B21] Malekzadeh F. Ghorbanzadeh M. S. Ghafourian H. Soudi M. R. (2007). Biosorption of tungstate by a bacillus sp. isolated from Anzali Lagoon. *World J. Microbiol. Biotechnol.* 23 905–910. 10.1007/s11274-006-9313-3

[B22] Milojevic T. Albu M. Blazevic A. Gumerova N. Konrad L. Cyran N. (2019). Nanoscale tungsten-microbial interface of the metal immobilizing thermoacidophilic archaeon *Metallosphaera sedula* cultivated with tungsten polyoxometalate. *Front. Microbiol.* 10:1267 10.3389/fmicb.2019.01267 PMC6593293 31275255

[B23] Mukherjee A. Wheaton G. H. Blum P. H. Kelly R. M. (2012). Uranium extremophily is an adaptive, rather than intrinsic, feature for extremely thermoacidophilic metallosphaera species. *Proc. Natl. Acad. Sci. U.S.A* 109 16702–16707. 10.1073/pnas.1210904109 23010932 PMC3478614

[B24] Oggerin M. Tornos F. Rodríguez N. del Moral C. Sánchez-Román M. Amils R. (2013). Specific jarosite biomineralization by *Purpureocillium lilacinum*, an acidophilic fungi isolated from río tinto. *Environ. Microbiol.* 8 2228–2237. 10.1111/1462-2920.12094 23425574

[B25] Ogi T. Makino T. Iskandar F. Tanabe E. Okuyama K. (2016). Heat-treated *Escherichia coli* as a high-capacity biosorbent for tungsten anions. *Bioresour. Technol.* 218 140–145. 10.1016/j.biortech.2016.06.076 27359063

[B26] Orange F. Disnar J.-R. Westall F. Prieur D. Baillif P. (2011). Metal cation binding by the hyperthermophilic microorganism, archaea *Methanocaldococcus jannaschii*, and its effects on silicification. *Palaeontology* 54 953–964. 10.5194/bgd-8-2235-2011

[B27] Orange F. Dupont S. Le Goff O. Bienvenu N. Disnar J.-R. Westall F. (2014). Experimental fossilization of the thermophilic gram-positive bacterium geobacillus SP7A: a long duration preservation study. *Geomicrobiol. J.* 31 578–589. 10.1080/01490451.2013.860208

[B28] Page K. Li J. Savinelli R. Szumila H. N. Zhang J. Stalick J. K. (2008). Reciprocal-space and real-space neutron investigation of nanostructured Mo2C and WC. *Solid State Sci.* 10 1499–1510. 10.1016/j.solidstatesciences.2008.03.018

[B29] Phoenix V. R. Martinez R. E. Konhauser K. O. Ferris F. G. (2002). Characterization and implications of the cell surface reactivity of calothrix sp. strain KC97. *Appl. Environ. Microbiol.* 68 4827–4834. 10.1128/AEM.68.10.4827-4834.2002 12324327 PMC126417

[B30] Rohwerder T. Gehrke T. Kinzler K. Sand W. (2003). Bioleaching review part a: progress in bioleaching: fundamentals and mechanisms of bacterial metal sulfide oxidation. *Appl. Microbiol. Biotechnol.* 63 239–248. 14566432 10.1007/s00253-003-1448-7

[B31] Sánchez-Román M. Puente-Sánchez F. Parro V. Amils R. (2015). Nucleation of Fe-rich phosphates and carbonates on microbial cells and exopolymeric substances. *Front. Microbiol.* 6:1024. 10.3389/fmicb.2015.01024 26441946 PMC4585095

[B32] Takashi O. Makino T. Okuyama K. Stark W. J. Iskandar F. (2016). Selective biosorption and recovery of tungsten from an urban mine and feasibility evaluation. *Ind. Eng. Chem. Res.* 55 2903–2910. 10.1021/acs.iecr.5b04843

[B33] Takashi O. Sakamoto Y. Nandiyanto A. B. D. Okuyama K. (2013). Biosorption of tungsten by *Escherichia coli* for an environmentally friendly recycling system. *Ind. Eng. Chem. Res.* 52 14441–14448. 10.1021/ie401193y

[B34] Veith A. Klingl A. Zolghadr B. Lauber K. Mentele R. Lottspeich F. (2009). Acidianus, sulfolobus and metallosphaera surface layers: structure, composition and gene expression. *Mol. Microbiol.* 73 58–72. 10.1111/j.1365-2958.2009.06746.x 19522740

[B35] Westall F. Cavalazzi B. (2011). “Biosignatures in rocks,” in *Encyclopedia of Geobiology*, ed. Reitner J., (Dordrecht: Springer).

[B36] Wheaton G. H. Mukherjee A. Kelly R. M. (2016). Transcriptomes of the extremely thermoacidophilic archaeon *Metallosphaera sedula* exposed to metal “Shock” reveal generic and specific metal responses. *Appl. Environ. Microbiol.* 82 4613–4627. 10.1128/AEM.01176-16 27208114 PMC4984275

[B37] Wheaton G. H. Vitko N. P. Counts J. A. Dulkis J. A. Podolsky I. Mukherjee A. (2019). Extremely thermoacidophilic metallosphaera species mediate mobilization and oxidation of vanadium and molybdenum oxides. *Appl. Environ. Microbiol.* 85:e02805-18. 10.1128/AEM.02805-18 30578261 PMC6384102

