# Supplementary material for: Biotransformation of Scheelite CaWO4 by the Extreme Thermoacidophile Metallosphaera sedula: Tungsten–Microbial Interface
Source: Front Microbiol. 2019 Jul 2;10:1492. doi: 10.3389/fmicb.2019.01492 (PMC6614383; doi:10.3389/fmicb.2019.01492)
Supplement: Supplementary file 1 [file Data_Sheet_1.PDF]

Supplementary Material

Supplementary Figures

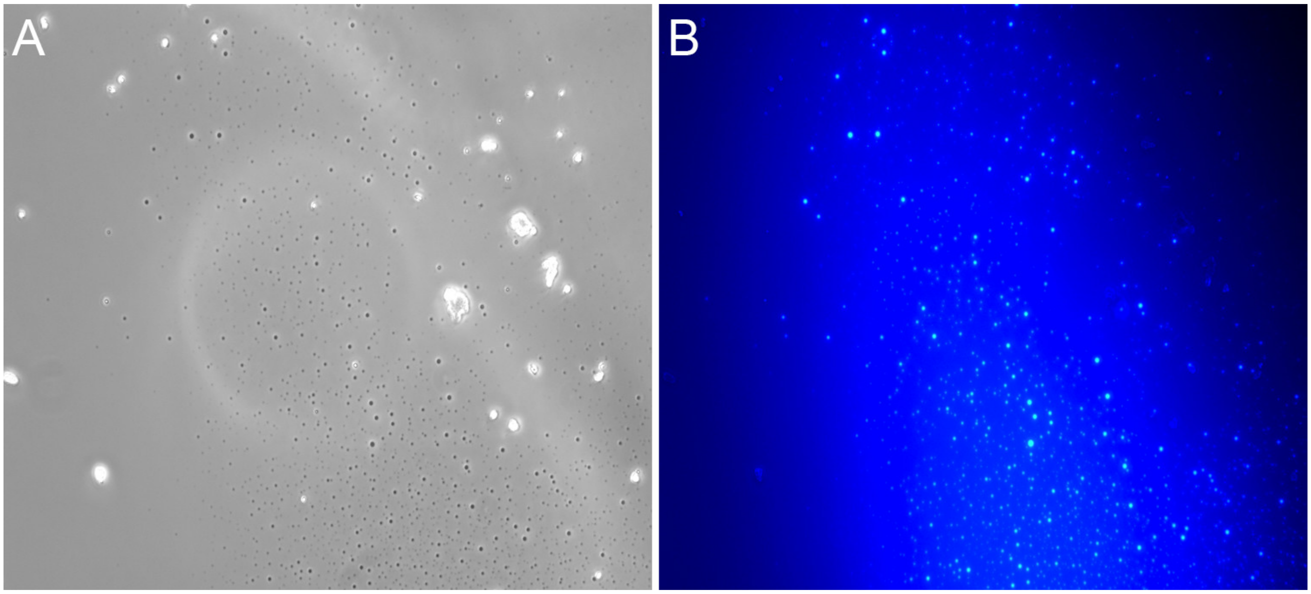

Supplementary Figure 1. Phase contrast and fluorescence micrographs of cells of *M. sedula* grown on scheelite as the sole energy source. Phase contrast (A) and fluorescence micrographs (DAPI stained) (B) of cells of *M. sedula* grown on scheelite at 73°C. Scale bar, 10 µm.

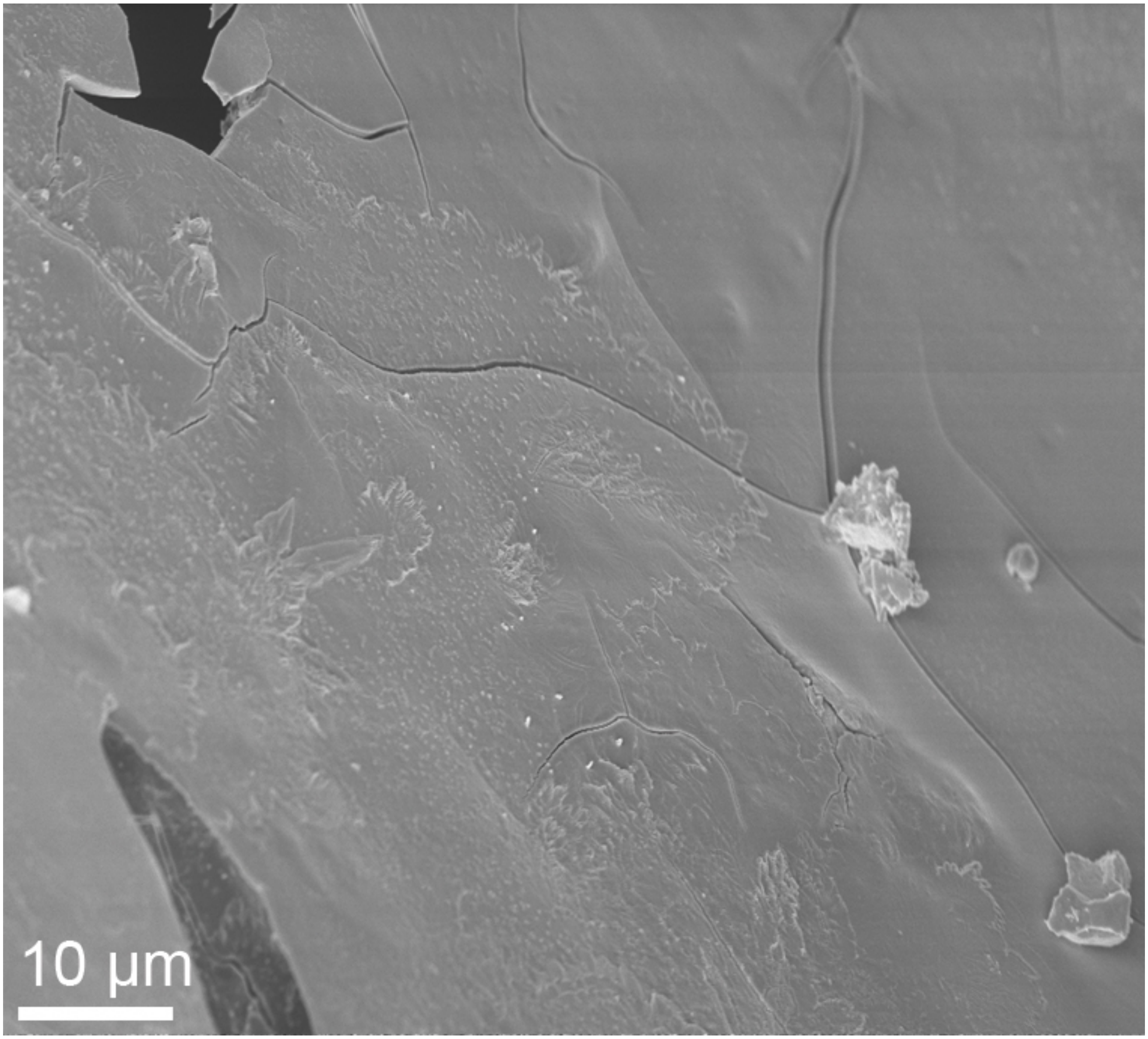

Supplementary Figure 2. Scanning electron microscopy (SEM) image of scheelite surface: surface of scheelite fragment abiotically incubated with culture medium without presence of *M. sedula* at 73°C.

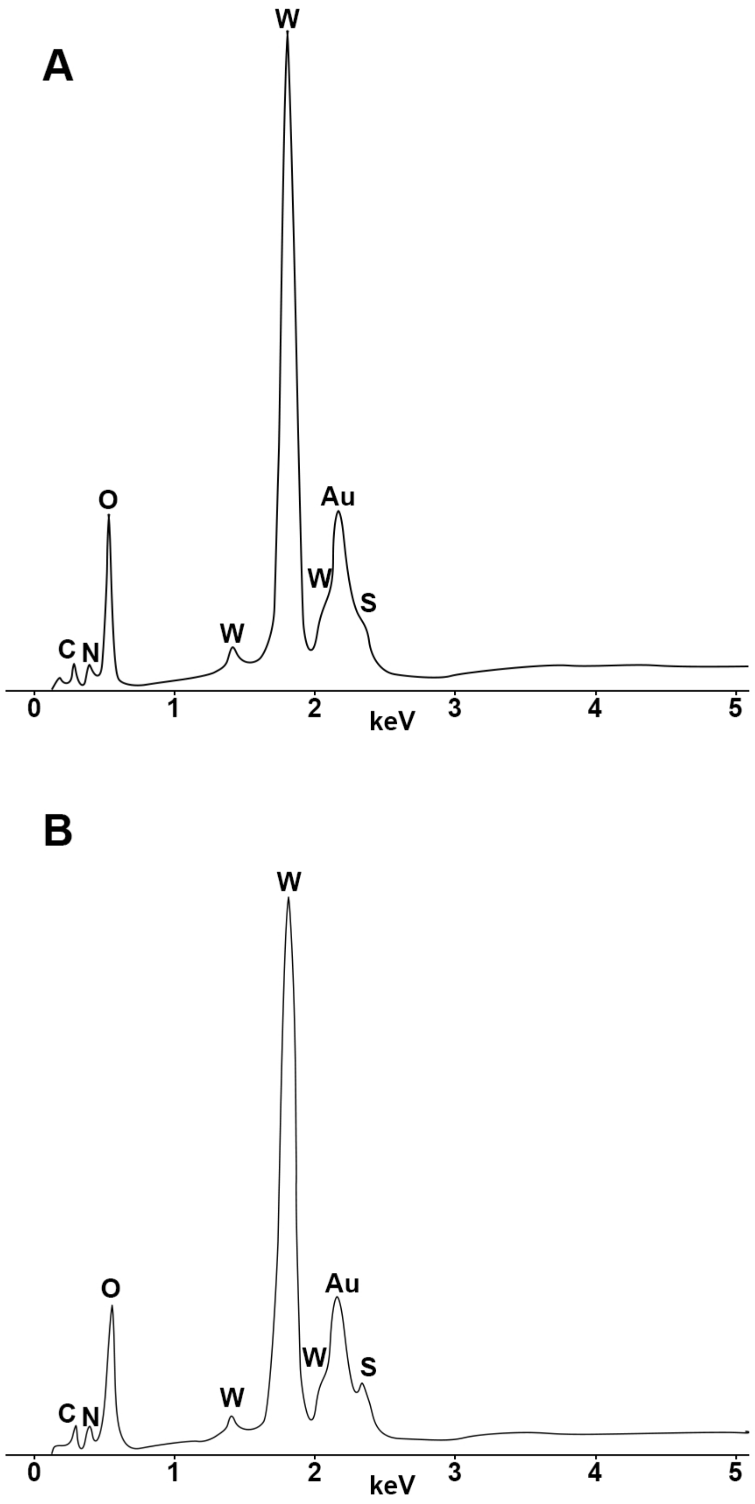

Supplementary Figure 3. Full length EDS spectra taken in marked regions of *M. sedula* cells grown on scheeilte and shown in Figure 4A. Au peaks in EDS spectra are due to sample coating with a Au layer.

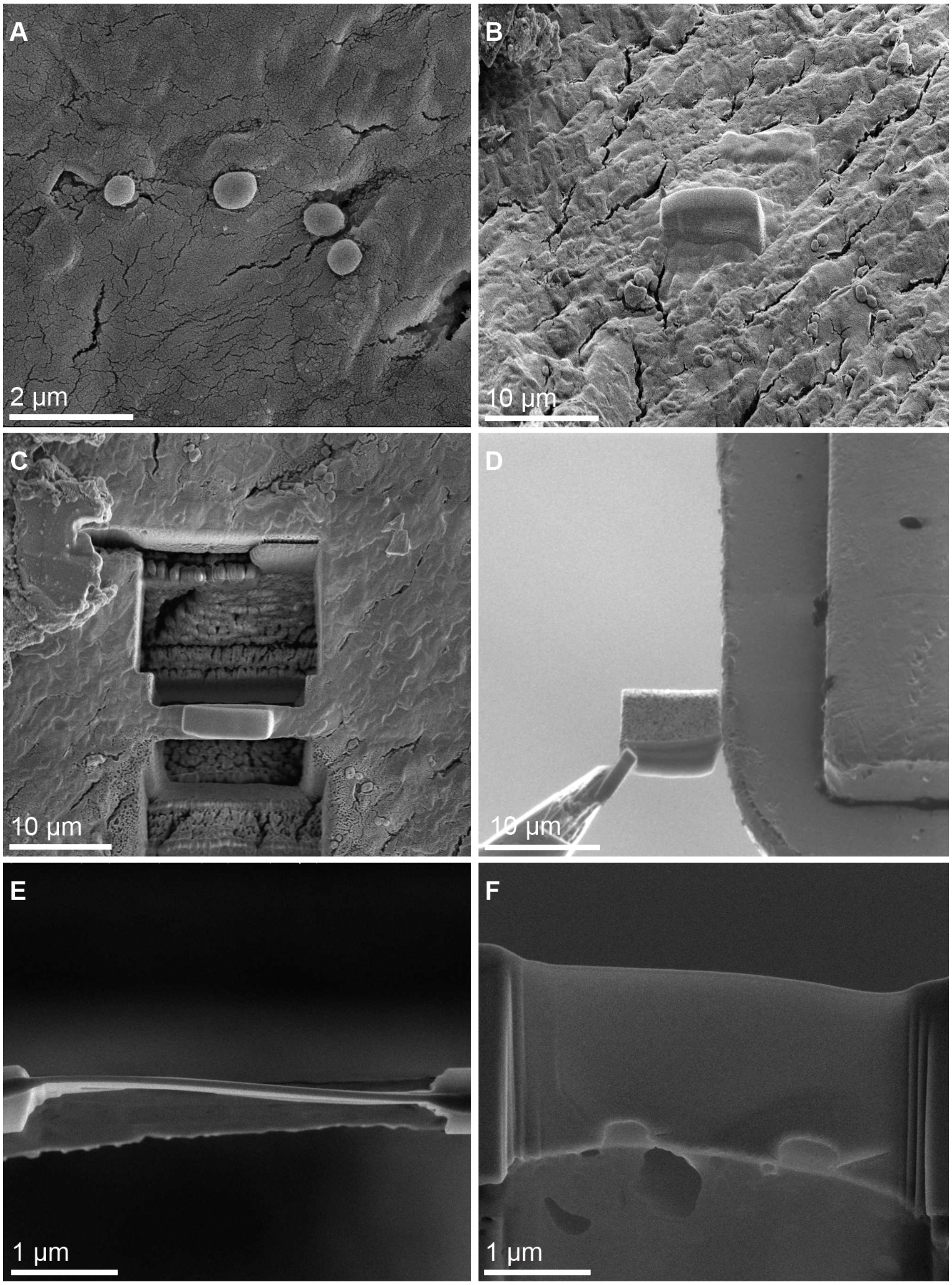

Supplementary Figure 4. Focused Ion Beam (FIB) assisted preparation of thin lamellae of *M. sedula* grown on scheelite documented by electron beam induced SEM images (A-C, E, F) and an ion beam induced SEM image (B). (A) SEM image of *M. sedula* cells attached to scheelite surface. (B) 3 µm thick Pt deposition layer covering cells of *M. sedula*. (C) FIB removal of material at both sides of the Pt layer viewed perpendicular to the substrate surface. (D) Transfer of the 2.5 µm thick lamella from the micromanipulator needle (left) to the Cu TEM grid (right). (E) Finally thinned lamella showing the Pt layer in top-view. (F) Side-view of the finally thinned lamella showing the two flattened cells attached to the substrate and covered by a Pt layer.

Video S1

Wiggling of *M. sedula* grown on scheelite as the sole energy source at 73°C after visualization by a modified DAPI fluorescence staining procedure. Recorded with Nikon microscope Jenoptik camera Nikon eclipse 50i microscope with ProgRes® MF cool camera.
